# LAMP2A regulates cisplatin resistance in colorectal cancer through mediating autophagy

**DOI:** 10.1007/s00432-024-05775-6

**Published:** 2024-05-08

**Authors:** Zhiliang Shi, Shuting Yang, Chenglong Shen, Jiazhe Shao, Fang Zhou, Haichen Liu, Guoqiang Zhou

**Affiliations:** 1 Department of Gastrointestinal Surgery, Affiliated Changshu Hospital to Nantong University, Changshu No. 2 Hospital, Changshu, 215500 Jiangsu Province China; 2 Department of Gastrointestinal Surgery, Affiliated Changshu Hospital to Nantong University, Changshu No. 2 Hospital, Suzhou, 215000 Jiangsu Province China

**Keywords:** Colorectal cancer, DDP, LAMP2A, Chemoresistance, Autophagy

## Abstract

**Background:**

Drug resistance is an important constraint on clinical outcomes in advanced cancers. LAMP2A is a limiting protein in molecular chaperone-mediated autophagy. This study was aimed to explore LAMP2A function in cisplatin (cis-diamminedichloroplatinum, DDP) resistance colorectal cancer (CRC) to seek new ideas for CRC clinical treatment.

**Methods:**

In this study, LAMP2A expression was analyzed by molecular experimental techniques,such as qRT-PCR and western blot. Then, LAMP2A in cells was interfered by cell transfection experiments. Subsequently, the function of LAMP2A on proliferation, migration, invasion, DDP sensitivity, and autophagy of CRC/DDP cells were further investigated by a series of experiments, such as CCK-8, transwell, and western blot.

**Results:**

We revealed that LAMP2A was clearly augmented in DDP-resistant CRC and was related to poor patient prognosis. Functionally, LAMP2A insertion remarkably CRC/DDP proliferation, migration, invasion ability and DDP resistance by strengthen autophagy. In contrast, LAMP2A knockdown limited the proliferation, migration, and invasion while heightened cellular sensitivity to DDP by restraining autophagy in CRC/DDP cells. Furthermore, LAMP2A silencing was able to curb tumor formation and enhance sensitivity to DDP in vivo.

**Conclusion:**

In summary, LAMP2A boosted malignant progression and DDP resistance in CRC/DDP cells through mediating autophagy. Clarifying LAMP2A function in DDP resistance is promising to seek cancer therapies biomarkers targeting LAMP2A activity.

## Introduction

Globally, colorectal cancer (CRC) is a commonly recognized causes of death due to cancer. New CRC cases are going toachieve 3.2 million worldwide by 2040 (Xi and Xu 2021). In recent decades, novel treatments in rapid development have dramatically reduced mortality in cancer patients (Siegel et al. 2021). However, advanced cancers clinical outcomes are still unsatisfactory. The survival rate (within 5 years) of CRC is still only about 50%. This low survival rate of CRC is highly related to its chemotherapy resistance (Ma et al. 2023). Therefore, elucidating the mechanism of chemoresistance in CRC is a hot research topic at home and abroad. Not to be ignored, acquired chemotherapeutic resistance can arise under therapeutic pressure, which confers acquired epigenetic alterations to cancer cells to enhance survival (Ming et al. 2023). In addition, the mechanisms that by which emerging resistance occurs are complex, and in addition to the regulation of gene transcription and post-transcriptional modifications, protein degradation has received growing focus for its immediate and fast regulation (Li et al. 2020a). Therefore, it is of great importance to continue to unravel the molecular mechanisms underlying the development of drug resistance in CRC and to seek possible biomarkers for the treatment of CRC drug resistance.

Autophagy is considered to be classified into macroautophagy (often referred to as autophagy), microautophagy, and molecular chaperone-mediated autophagy (CMA) (Glick et al. 2010). The major distinction for CMA from other types of autophagy (e.g., macroautophagy and microautophagy) is that it optionally targets and degrades particular substrate proteins while not influencing the organelle or neighboring proteins (Li et al. 2020b). LAMP2A, located mainly in lysosomal membranes, is a key factor in and contributes significantly to CMA (Kiffin et al. 2004). During CMA, LAMP2a binds target proteins in conjunction with its chaperone protein HSC70, and then forms homodimers to transport the target proteins into the lysosome to “selectively” remove the target proteins without disturbing neighboring proteins (Bandyopadhyay et al. 2008).

Lately, a growing fascination with how LAMP2A affects the genesis and development of cancer cells. Some reports have revealed that LAMP2A is increased in tumor and plays a pro-tumorigenic role (Qiao et al. 2023). For example, in breast cancer, LAMP2A expression in tumor-associated macrophages correlates with poor prognosis. LAMP2a promotes tumor progression by regulating macrophage activation and function, which is specifically mediated through the degradation of PRDX1 and CRTC1 (Wang et al. 2019). LAMP2A expression is dramatically elevated in CRC patients and mouse models, and suppression of CMA by LAMP2A silencing promotes apoptosis and impedes proliferation of CT26 cells (Peng et al. 2020). LAMP2A overexpression was presented in clinical samples of glioblastoma (GBM) tissues and resulted in higher grade and poorer overall survival (Auzmendi-Iriarte et al. 2022). However, another study reported opposite findings that in human hepatocellular carcinoma (HCC), LAMP2A was reduced and LAMP2A silencing facilitated HCC cell progression (Desideri et al. 2023). These studies suggest a growing link between LAMP2A and cancer biology. However, the exact role of LAMP2A in CRC drug resistance remains unclear.

In this study, we worked to elucidate the function of LAMP2A in CRC drug resistance and assess its potential in overcoming cisplatin (cis-diamminedichloroplatinum, DDP)-resistant therapy. We revealed that LAMP2A was augmented in CRC tissues and was related to DDP resistance in CRC. Furthermore, LAMP2A strengthened proliferation, migration, invasion and DDP resistance of CRC/DDP cells by mediating autophagy. These results provide strong evidence for the development of cancer therapies targeting LAMP2A activity.

## Methods

### Tissue sample collection

All experiments involving the studied patient samples were authorized by the Changshu NO.2 People's Hospital Ethics Committee. Clinical tissue specimens were obtained during surgery and diagnosed as CRC tissues by histopathology after full informed consent were given from all human subjects under study. In addition, CRC tissues were further classified into DDP-sensitive and DDP-resistant tissues based on the patient's treatment response to DDP.

### Cell culture

All cell lines were cultured using RPMI-1640 complete medium (containing 10% FBS), including FHC, SW480, SW480/DDP, HCT116, HCT116/DDP, HT29, RKO, and SW620. Among them, FHC, SW480, HCT116, HT29, RKO, and SW620 cell lines were obtained from the Chinese Academy of Sciences cell bank. SW480/DDP was purchased from Meixuan (Shanghai). HCT116/DDP cells were purchased from Fenghui (Changsha). Then, all cells grew in a constant temperature incubator at 37 °C with 5% CO_2_. In addition, 2 μM DDP (sigama) was added to the medium of CRC/DDP cells to maintain the DDP-resistant phenotype, and transferred to DDP-free medium for 1 week before use.

### qRT-PCR

Total RNA was extracted from using an RNA extraction kit (Tiangen) according to each manufacturer's protocol. cDNA was then reverse transcribed from RNA using a reverse transcription kit (Prime Script RT Kit, Takara). GAPDH was applied as an internal reference to standardize the target gene expression and analyzed by the 2^–ΔΔCt^method. Primer information was detailed in Table 1.

**Table 1:** primers for qRT-PCR

Gene	Primers sequences (5’–3’)
LAMP2A	F: CTCTGCGGGGTCATGGTG R: CGCACAGCTCCCAGGACT
GAPDH	F: GTCGGTGTGAACGGATTTG R: TCCCATTCTCAGCCTTGAC

### Western blot

For total cellular protein extraction, cells were collected and RIPA buffer (Beyotime) was applied to lyse cells. The extracted proteins were then quantified by BCA protein concentration assay kit (Beyotime). Subsequently, proteins were separated utilizing 10% SDS-PAGE and transferred to a PVDF membrane (Millipore). Next, the membranes were closed for 1 h at room temperature using 5% skim milk. The membranes were incubated with various primary antibodies overnight at 4 °C, using GAPDH, LAMP2A, p62, and LC3II/LC3I as primary antibodies. The next day, primary antibodies were recovered and membranes were continued incubating using secondary antibodies for 1 h. Finally, protein bands were visualized by ECL kit (Millipore). Image J software was used for gray scale analysis of protein bands and calculated as relative expression of GAPDH.

### Cell transfection

Targeted LAMP2A-shRNA lentiviral vectors, overexpression vectors and corresponding empty vector or plasmid were purchased from Genepharma. Cells (3 × 10^5^cells/ml per well) were inoculated in 6-well plates and cell transfection was performed when the cell density reached 60–70%. Cells were infected with LAMP2A-shRNA lentiviral vector/overexpression plasmid and controls, respectively, while ploybrene (6 μg/ml, sigama) was added to enhance the transfection efficiency. The medium was changed to fresh complete medium after 8 h of infection. After continuing the culture for 48 h, the stably transfected cells were screened with 2 μg/ml puromycin (sigama). And green fluorescent protein expression was evaluated for overexpressing LAMP2A cells by fluorescence microscope. Finally, qRT-PCR and western blot was employed to analyze the knockdown or overexpression transfection efficiency.

### CCK-8

Cell proliferation was detected by CCK-8 kit (Dojindo). Knockdown or overexpression cells were inoculated in 96-well plates. Then, 10 μL of CCK-8 solution was added after 0, 24, 48, 72 h of incubation. Subsequently, the plates were further incubated at 37 °C for 2 h. Finally, the absorbance of the cells at 450 nm was measured to assess the cell proliferation ability.

### IC50 assay

Cells (8000/well) were spread in 96-well plates, and when the cell density reached 70–80%, a gradient concentration of DDP (0, 10, 20, 40, 60, 80, 100, 120 μM) was added to continue the incubation for 48 h. Then, the IC50 was detected based on cell viability using CCK-8.

### Transwell assays for cell migration and invasion

Cell migration ability was assessed using Transwell chambers (8 μm, BD Biosciences). Knockdown or overexpressed cells (serum-free medium) were directly inoculated in the upper chamber and 600 μL of complete medium (with 10% FBS) was added to the lower chamber. Subsequently, the cells were further incubated at 37 °C for 24 h after which the upper chamber was wiped off. Cells in the lower chamber were fixed through 4% paraformaldehyde and stained with 1% crystal violet. Finally, five randomly selected fields of view were counted and photographed. For cell invasion, after matrix gel (sigama) pretreated with upper chambers, other procedures were the same as above.

### Animal experiments

The animal procedures involved in this study were operated with the approval of Xuzhou Medical University Animal Care and Use Committee. 4–6-week-old male Balb/c nude mice were obtained from Vital River Laboratory (Beijing). The nude mice were randomly divided into 4 groups (PBS + sh-NC group, PBS + sh-LAMP2A group, DDP + sh-NC group, and DDP + sh-LAMP2A group), with 3 mice in each group. SW480 cells (1 × 10^7^cells/mouse) of sh-LAMP2A or sh-NC were inoculated in right flank of nude mice to construct tumor models. Then, drug treatment was performed according to subgroups. Nude mice in the DDP group were given 4 mg/kg/d of DDP weekly by intraperitoneal injection (100 μl/d PBS was given in the PBS group). Meanwhile, the volume was detected every 4 days, and the volume was calculated by the following formula: volume = length × width × width^2^× 0.5. On the 16th day, the mice were euthanized and the subcutaneous tumors were collected and weighed. In addition, subcutaneous tumor tissues were embedded by paraffin and stained by immunohistochemistry (IHC) to detect the expression of LAMP2A.

### Statistical analysis

Data were statistically analyzed using GraphPad Prism. All cellular experiments were expressed as the mean ± SD of three independent experiments. Unpaired Student's t-test was employed to analyze the results of two different sets of experiments. Statistically significant results were indicated as *p*< 0.05.

## Results

### LAMP2A is upregulated in DDP resistant CRC tissues

**Fig. 1:**
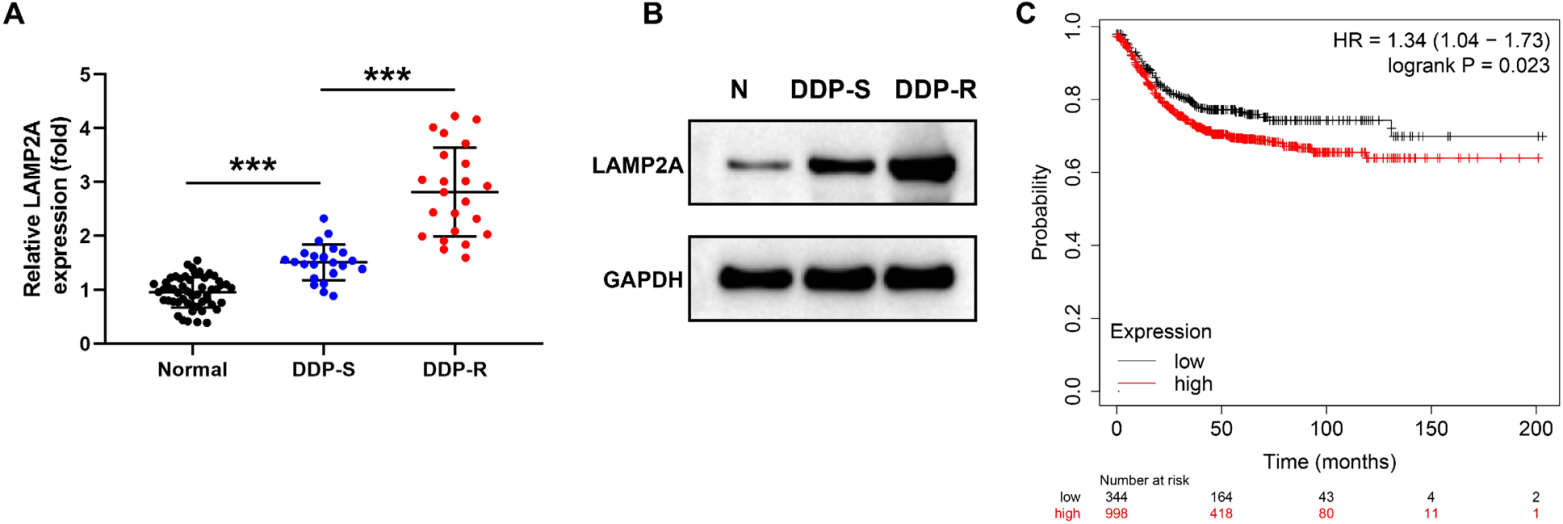
LAMP2A was upregulated in resistant CRC tissues and associated with poor prognosis. **A** The mRNA level of LAMP2A in normal tissues (n = 52), DDP sensitive CRC tissues (n = 23) and DDP resistant CRC tissues (n = 23). **B** Representative protein expression of LAMP2A in normal tissue and CRC tissues. **C** Survival curves of CRC patients with high expression and low expression of LAMP2 was analyzed using Kaplan–Meier Plotter database. ***P< 0.001

To explore the role of LAMP2A, we examined its expression firstly. We discovered that the expression of LAMP2A mRNA was 2.26-fold higher in CRC tissues than in normal tissues. Further to investigate whether LAMP2A was linked to DDP resistance in CRC. We categorized CRC clinical samples into DDP-resistant (DDP-R) and DDP-sensitive (DDP-S). The results showed that LAMP2A was increased 0.86-fold in the tissues of DDP-resistant patients compared with DDP-sensitive patients (Fig. 1A). Similarly, western blot results demonstrated the same trend of LAMP2A protein expression (Fig. 1B). In addition, Kaplan–Meier analysis displayed that patients with high expression of LAMP2A had relatively lower survival rates (*P*< 0.05) (Fig. 1C). The above results demonstrated that LAMP2A was implicated in the development of DDP resistance in CRC and was linked to poor patient prognosis. Next, in order to evaluate the pathological significance of LAMP2A in CRC tissue expression, we analyzed the clinical and pathological characteristics of the patient. The findings displayed that LAMP2A was obviously associated with tumor differentiation, tumor stage, tumor size, and lymph node metastasis, while the expression of LAMP2A was not significantly related to age and sex (Table 2).

**Table 2:** Correlations between LAMP2A and clinical characteristics of 46 CRC patients

Characteristics	Total Number	LAMP2A expression		
		Low(n = 23)	High(n = 23)	*P*value
Age	46			> 0.9999
≤ 60		11	12	
> 60		12	11	
Sex	46			> 0.9999
Male		10	11	
Female		13	12	
Differentiation	46			0.0156*
Low		18	9	
High		5	14	
Tumor stage	46			0.0169*
I + II		15	6	
III + IV		8	17	
Tumor size (cm)	46			0.0377*
≤ 5		16	8	
> 5		7	15	
Lymph node metastasis	46			0.0018**
No		14	3	
Yes		9	20	

### LAMP2A is raised in DDP resistant CRC cell lines

**Fig. 2:**
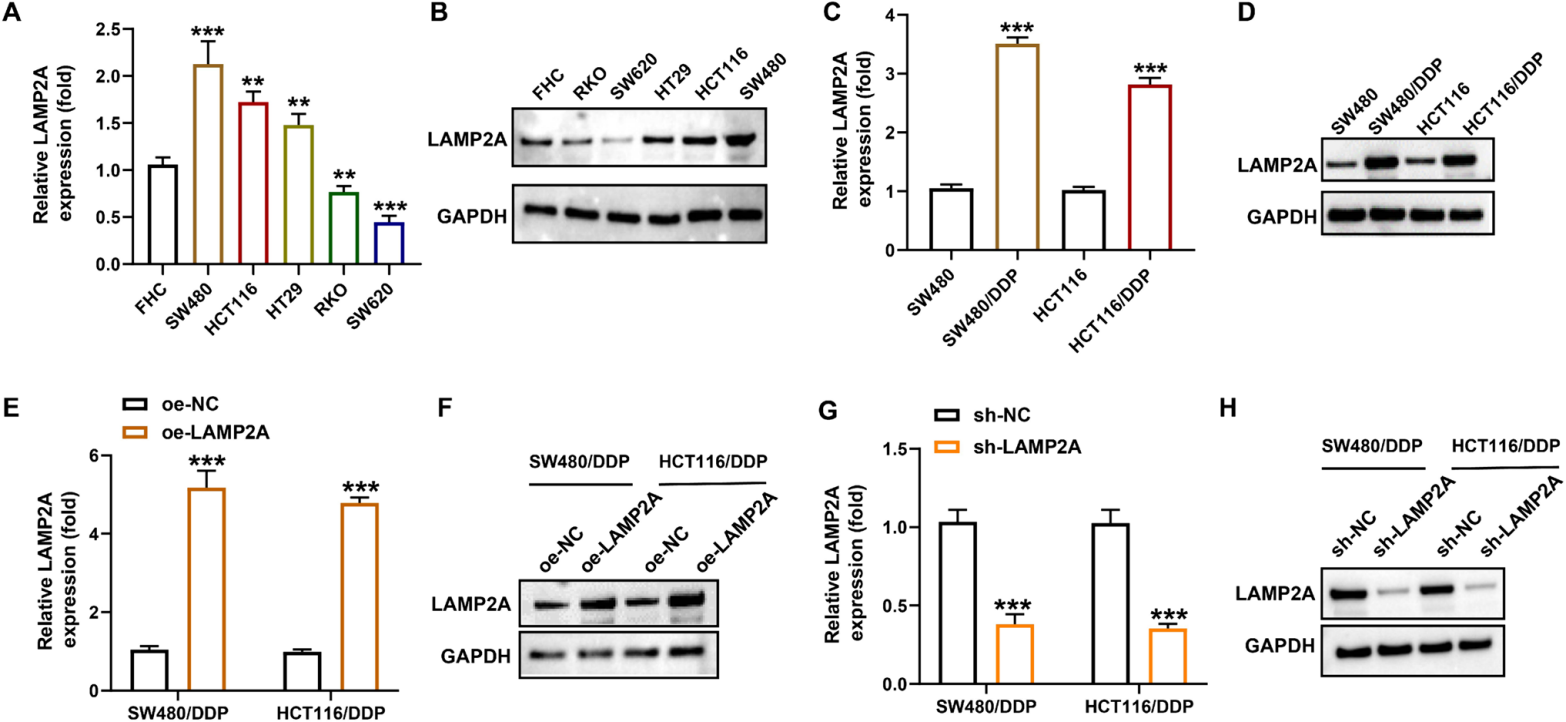
LAMP2A was raised in DDP resistant CRC cell lines. **A** The mRNA expression of LAMP2A in different CRC cell lines was detected by qRT-PCR. **B** The protein level of LAMP2A in different CRC cell lines was detected by western blot. **C** The mRNA expression of LAMP2A in SW480/DDP and HCT116/DDP cells. **D** The protein expression of LAMP2A in SW480/DDP and HCT116/DDP cells. **E**–**F** Overexpression efficiency in SW480/DDP and HCT116/DDP cells. **G**–**H** Knockdown efficiency in SW480/DDP and HCT116/DDP cells. **P< 0.01, ***P< 0.001

Next, we verified whether LAMP2A expression in CRC cells was consistent with that in tissues. We found that compared to normal cells FHC, LAMP2A (both the mRNA and protein expression) were clearly improved in some of the CRC cells (SW480, HCT116, HT29) (1.39–2.0 fold). SW480 and HCT116 with the highest expression were selected for the subsequent study (Fig. 2A and B). Next, LAMP2A expression in CRC/DDP cells was verified. As expected, LAMP2A mRNA in SW480/DDP and HCT116/DDP cells was 2 and 1.63-fold higher than that in SW480 and HCT116 cells (Fig. 2C). The LAMP2A protein expression showed a similar trend (Fig. 2D). Subsequently, to investigate the specific function of LAMP2A in CRC drug resistance, we interfered with LAMP2A levels in cells by cell transfection assay. As shown in Fig. 2E–H, the expression (mRNA and protein) of LAMP2A was distinctly strengthened after transfection with the overexpression plasmid (Fig. 2E and F). Whereas, the expression (mRNA and protein) of LAMP2A was remarkably limited after sh-RNA transfection (Fig. 2G and H). The above results revealed that LAMP2A may play a key role in the process of DDP resistance in CRC.

### Overexpression of LAMP2A enhances malignant progression and DDP resistance in CRC/DDP cells by promoting autophagy

**Fig. 3:**
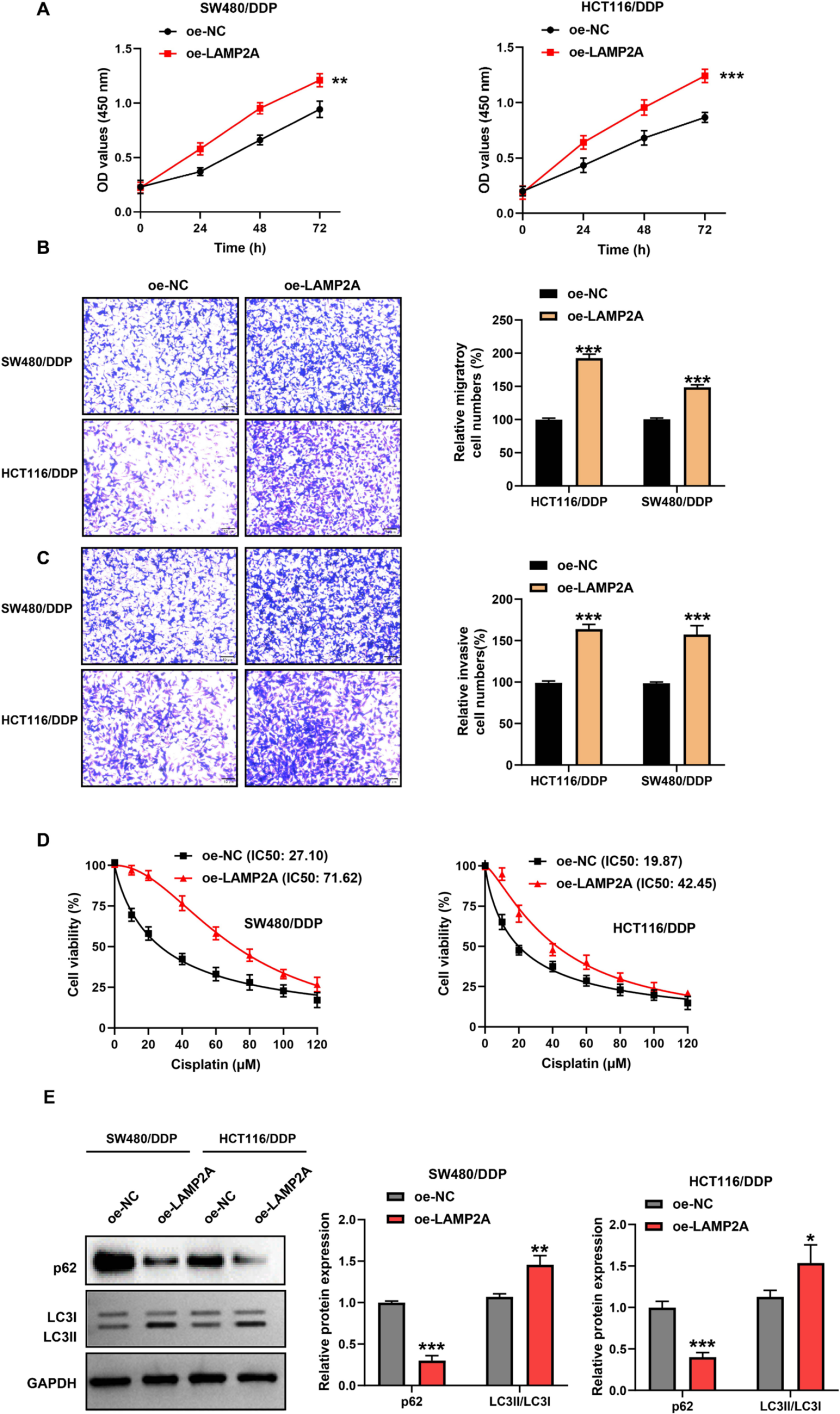
LAMP2A overexpression promoted the malignancy and increased DDP resistant in CRC cells. **A** Cell proliferation was assessed by CCK8 assay. **B** Cell migration was detected by transwell assay. **C** Cell invasion was detected by transwell assay. **D** IC50 of transfected cells was assessed by CCK8 assay. **E** LC3 and p62 protein expression was measured by western blot. *P< 0.05, **P< 0.01, ***P< 0.001

Subsequently, we further investigated the effects of overexpression of LAMP2A on the proliferation, migration, invasion, DDP sensitivity, and autophagy of CRC/DDP cells through a series of assays, such as CCK-8, transwell, and western blot. The outcomes demonstrated that after 72h of cell culture, the proliferation capacity of SW480/DDP and HCT116/DDP cells after overexpression of LAMP2A was 1.28-fold and 1.43-fold higher than that of the control, respectively (Fig. 3A). Similarly, the cell migration (1.48 and 1.93-fold) and invasion (1.60 and 1.65-fold) were also highly enhanced upon LAMP2A overexpression (Fig. 3B and C). Next, we explored whether LAMP2A overexpression would have an effect on cellular DDP sensitivity. Frustratingly, LAMP2A insertion distinctly restrained the sensitivity of CRC cells to DDP (Fig. 3D). Finally, we investigated the effect of LAMP2A overexpression on intracellular autophagy. We found that the expression of p62, a key protein for autophagy, was curbed, while the LC3II/LC3I ratio was augmented. The p62 in the control group was 3.32 and 2.50-fold higher than that in the LAMP2A introduction group, whereas the LC3II/LC3I ratio in the overexpression of LAMP2A group was 1.35 and 1.36-fold higher than that in the control group, which suggesting that cellular autophagy was promoted (Fig. 3E). However, addition of the autophagy inhibitor 3-MA reversed the effects of LAMP2A overexpression on autophagy-associated proteins (Figure S1). In summary, LAMP2A overexpression strengthened the proliferation, migration, invasion ability and DDP resistance of CRC/DDP by stimulating autophagy.

### Knockdown of LAMP2A curbs malignant progression and DDP resistance in CRC/DDP cells through inhibition of autophagy

**Fig. 4:**
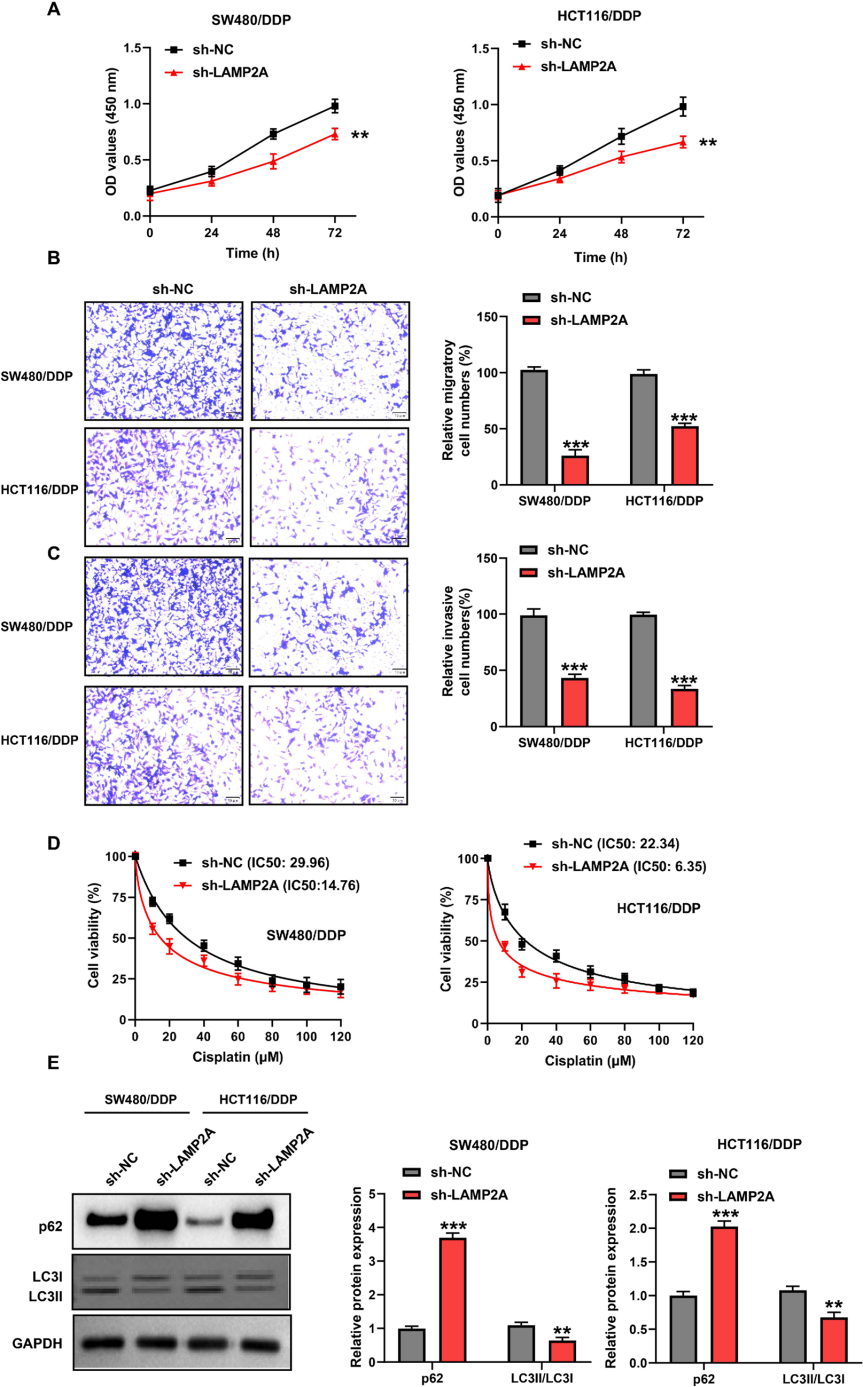
LAMP2A knockdown inhibited the malignancy and increased DDP sensitivity in CRC cells. **A** Cell proliferation was assessed by CCK8 assay. **B** Cell migration was detected by transwell assay. **C** Cell invasion was detected by transwell assay. **D** IC50 of transfected cells was assessed by CCK8 assay. **E** LC3 and p62 protein expression was measured by western blot. **P< 0.01, ***P< 0.001

Next, we similarly study the function of LAMP2A knockdown. In contrast to the LAMP2A overexpression results, we saw a series of better directions of development. For example, when knockdown intracellular LAMP2A expression, the proliferative capacity of cells was significantly reduced. The proliferative capacity of SW480/DDP and HCT116/DDP cells in the sh-NC group was 1.34 and 1.47 times larger than that in the sh-LAMP2A group, respectively (Fig. 4A). Similarly, the migratory (3.96 and 2.29-fold) and invasive (1.89 and 2.97-fold) abilities of cells in the sh-NC group were markedly greater than those in the sh-LAMP2A group (Fig. 4B and C). In addition, LAMP2A silencing limited DDP resistance in CRC cells (Fig. 4D). Finally, we also found that LAMP2A silencing enhanced p62 accumulation and instead restrained the LC3II/LC3I ratio, suggesting that cellular autophagy was also suppressed (Fig. 4E). In summary, LAMP2A knockdown lightened the proliferation, migration, and invasion ability of CRC/DDP and heightened the sensitivity of cells to DDP by alleviating autophagy.

### Knockdown of LAMP2A inhibits CRC tumor formation and DDP resistance in vivo

**Fig. 5:**
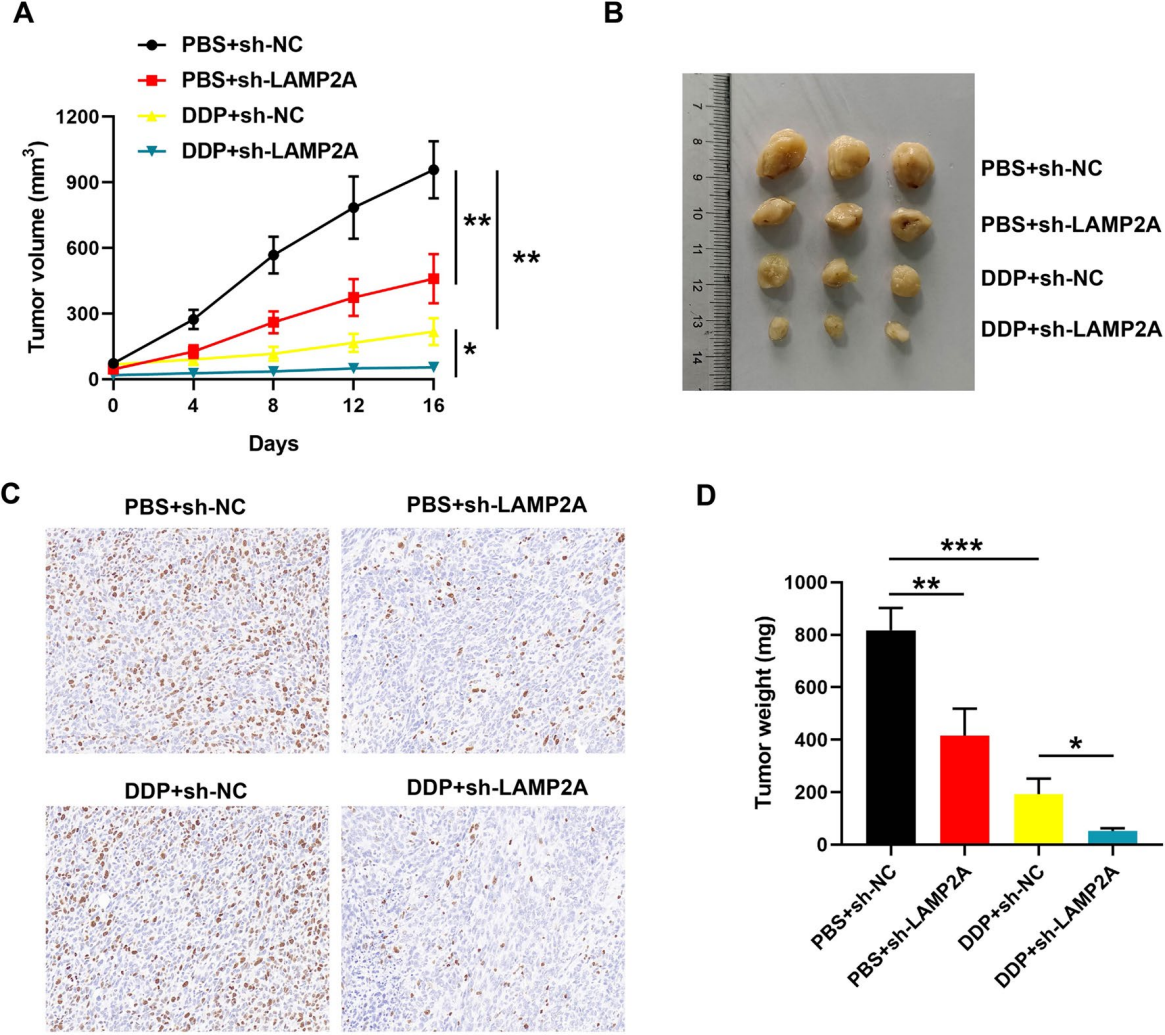
LAMP2A knockdown enhanced DDP efficacy against to CRC in vivo. LAMP2A knockdown SW480/DDP cells were implanted subcutaneously into the right flank of nude mice. DDP (4 mg/kg) was administered intraperitoneally per week in DDP treatment groups, and equal amount PBS was administered in control group. **A** Tumor volume was measured every week. **B** The representative tumor image of different groups. **C** The expression of LAMP2A was detected by immunohistochemical staining. **D** Tumor weight was measured after sacrificed. *P< 0.05, **P< 0.01, ***P< 0.001

Finally, we verified the anti-tumor effect of LAMP2A silencing in vivo by animal experiments. The findings displayed that after 16 days, the subcutaneous tumor volume and weight in the PBS + sh-NC group were 2.09 and 1.97 fold higher than those in the PBS + sh-LAMP2A group, respectively. Furthermore, the subcutaneous tumor volume and weight in the DDP + sh-NC group was 3.96 and 3.77 times fold higher than those in the DDP + sh-LAMP2A group, respectively (Fig. 5A and D). A representative image of the tumor was shown in Fig. 5B. This indicated that LAMP2A silencing weakened the tumor formation ability of mice and augmented the sensitivity of nude mice to DDP treatment. Finally, we assessed LAMP2A expression in subcutaneous tumor tissues by IHC. We found that LAMP2A was highly reduced in PBS + sh-LAMP2A and DDP + sh-LAMP2A group (Fig. 5C). In summary, knockdown of LAMP2A was able to restrain tumor formation and strengthened the sensitivity of the organism to DDP in vivo.

## Discussion

Metastasis is a major challenge in cancer treatment (Khan et al. 2023). Autophagy has the functions of maintaining intracellular homeostasis, regulating cellular differentiation and responding to external cellular stress. The autophagy-lysosome pathway is associated with a variety of cancer traits, such as death resistance, evasion of immune surveillance, and metabolic dysregulation, and plays an important role in a wide range of human diseases (Wani et al. 2021b). In the process of autophagy, pathways such as AKT (Wani et al. 2019), AMPK (Wani et al. 2021a), and mTOR (Khan et al. 2022) play important roles. CMA is implicated in many bio-processes, including transcriptional regulation, DNA replication, and immune response regulation. In particular, numerous investigations have reported that CMA often plays a pro-cancer function in tumor progression (Kaushik and Cuervo 2018). The reason for this may be due to two points; on the one hand, CMA-degrading proteins can provide tumor cells with the energy they need for growth. On the other hand, CMA-degrading proteins can provide raw materials for biosynthesis in tumor cells (Zhou et al. 2016; Xie et al. 2015). There is a rate-limiting step in CMA where the substrate binds to LAMP2A (Cuervo and Dice 2000). The expression and activity of LAMP2A directly affects the activity of CMA, and thus targeting LAMP2A may enable the targeting of CMA in the clinic.

It has been documented that LAMP2A is highly exhibited in a number of malignant tumors, such as HCC cells, lung cancer cells, gastric cancer cells, and cervical cancer cells (Kon et al. 2011). For example, it was found that high LAMP2A in breast cancer contributed to poor prognosis and increased cancer cell viability in HER2-negative breast cancer patients (Tokarchuk et al. 2021). Similarly, in non-small cell lung cancer, elevated LAMP2A expression was also observed, which contributed to the poor prognosis of patients and DDP resistance development. In contrast, knockdown of LAMP2A expression improved therapeutic properties and sensitivity to DDP treatment in mice (Ichikawa et al. 2020). In addition, it has been reported that upregulated LAMP2A-mediated hyperactivation of CMA in human grade IV glioblastoma regulates malignant progression of tumor cells by targeting SMAD3 degradation (Liu et al. 2022). Upregulation of LAMP2A expression also regulates apoptosis and proliferation of CRC cells (Peng et al. 2020). However, there are still few studies on LAMP2A in CRC progression, especially reports related to chemoresistance. Only one report was found that high LAMP2A led to 5-FU resistance and enhanced PLD2 through NF-κB pathway activation, thereby promoting the proliferation, invasion, and anti-apoptotic functions of CRC drug-resistant cells (Xuan et al. 2021). Therefore, more investigations are required to reveal the influence and mechanism of LAMP2A on CRC resistant to different chemotherapeutic agents. Consistent with the above reports, LAMP2A expression was also enhanced in CRC tissues, and high levels of LAMP2A were linked to poor prognosis in CRC. In addition, we found that LAMP2A was obviously augmented in DDP-resistant CRC as well, suggesting that LAMP2A may be associated with DDP-resistant progression in CRC. Furthermore, LAMP2A overexpression heightened CRC/DDP progression and DDP resistance. In contrast, LAMP2A knockdown lightened the malignant progression of CRC/DDP and increased cell sensitivity to DDP.

DDP is one of the platinum-based chemotherapeutic agents used to treat various types of cancer (Song et al. 2022; Shen et al. 2022). During DDP chemotherapy, the drug forms a complex with deoxyribonucleic acid (DNA), which inhibits the formation of ribonucleic acid from DNA and thus induces apoptosis (Dasari and Tchounwou 2014). Although the majority of patients have a good initial response to DDP, most patients eventually relapse and progress. This is due to the development of resistance to DDP in this group of patients (Wang et al. 2021). This resistance constrains the prognosis of CRC patients and makes the cancer progress in a bad direction (Makovec 2019). Therefore, it is important to explore the mechanism of DDP resistance in CRC to improve the treatment of CRC patients. More and more studies have shown that autophagy is closely related to chemotherapy resistance. Autophagy plays a dual role in the progression of drug resistance in cancer (Sui et al. 2013). Several reports have confirmed that various antitumor agents induce autophagic death of cancer cells (Liu et al. 2017). However, there is also evidence that autophagy contributes to chemotherapy resistance in various types of cancer (Luo et al. 2021). In addition, autophagy is often accompanied by changes in associated marker proteins. p62 is involved in intracellular signaling and regulation of autophagy, which in turn maintains cellular homeostasis (Vargas et al. 2023). Impaired or inhibited cellular autophagy leads to the accumulation of p62, which in turn activates intracellular signaling pathways associated with the p62 protein (Lamark et al. 2017). Furthermore, LC3 protein is another signature protein associated with autophagy. LC3II expression shows a positive correlation with autophagic activity (Peña-Martinez et al. 2022). LC3II protein induces the fusion of autophagosomes with lysosomes to accomplish the degradation of damaged mitochondria (Heckmann and Green 2019). In addition, LC3II is converted from LC3I, so an increase in LC3II is often accompanied by a decrease in LC3I (Mizushima and Yoshimori 2007). Therefore, LC3II/LC3I is also used as an indicator to characterize autophagic activity. We found that when intracellular LAMP2A was enhanced, p62 was limited, and the ratio of LC3II/LC3I was enhanced, which indicated that autophagy was strengthened in cells. In contrast, when the intracellular LAMP2A was attenuated, the accumulation of p62 was induced, while the ratio of LC3II/LC3I was restrained, which indicated that autophagy was weakened in the cell. This revealed that LAMP2A motivated the proliferation, migration, invasion and DDP resistance of CRC/DDP cells by mediating autophagy. Finally, in vivo experiments also confirmed that knockdown of LAMP2A limited tumor formation and boosted the sensitivity of the organism to DDP. In summary, this study established that LAMP2A mediated cisplatin resistance in CRC by stimulating autophagy. However, there are still many questions to be solved and refined in our study. For example, does LAMP2A also affect chemoresistance in CRC through other signaling pathways? In addition, the upstream and downstream regulatory mechanisms by which LAMP2A affects autophagy still need to be further investigated. These queries will continue to be investigated in depth in future studies.

## Conclusion

LAMP2A boosted CRC progression and DDP resistance by mediating autophagy. This study clarifies the potential of LAMP2A in overcoming CRC DDP-resistant therapy and may provide some theoretical basis for the development of cancer therapies targeting LAMP2A mediated-autophagy activity.

## Data Availability

The data are available from the corresponding author on reasonable request.
